# Development and Application of a Next Generation Air Sensor Network for the Hong Kong Marathon 2015 Air Quality Monitoring

**DOI:** 10.3390/s16020211

**Published:** 2016-02-05

**Authors:** Li Sun, Ka Chun Wong, Peng Wei, Sheng Ye, Hao Huang, Fenhuan Yang, Dane Westerdahl, Peter K.K. Louie, Connie W.Y. Luk, Zhi Ning

**Affiliations:** 1 School of Energy and Environment, City University of Hong Kong, Tat Chee Avenue, Kowloon, Hong Kong, China; lisun4@cityu.edu.hk(L.S.); electronic_joe@yahoo.com.hk (K.C.W.); wp5621679@gmail.com (P.W.); ttllttttlltt@gmail.com (S.Y.); huang1989hao@gmail.com (H.H.); fhyang2012@gmail.com (F.Y.); danewest03@gmail.com (D.W.); 2 Guy Carpenter Climate Change Centre, City University of Hong Kong, Tat Chee Avenue, Kowloon, Hong Kong, China; 3 Environmental Protection Department, the Government of the Hong Kong Special Administration Region, 33/F Revenue Tower, 5 Gloucester Road, Wan Chai, Hong Kong, China; plouie@epd.gov.hk (P.K.K.L.); conniechow@epd.gov.hk (C.W.Y.L.)

**Keywords:** marathon, next generation sensor, air network, roadside air quality

## Abstract

This study presents the development and evaluation of a next generation air monitoring system with both laboratory and field tests. A multi-parameter algorithm was used to correct for the impact of environmental conditions on the electrochemical sensors for carbon monoxide (CO) and nitrogen dioxide (NO_2_) pollutants. The field evaluation in an urban roadside environment in comparison to designated monitors showed good agreement with measurement error within 5% of the pollutant concentrations. Multiple sets of the developed system were then deployed in the Hong Kong Marathon 2015 forming a sensor-based network along the marathon route. Real-time air pollution concentration data were wirelessly transmitted and the Air Quality Health Index (AQHI) for the Green Marathon was calculated, which were broadcast to the public on an hourly basis. The route-specific sensor network showed somewhat different pollutant patterns than routine air monitoring, indicating the immediate impact of traffic control during the marathon on the roadside air quality. The study is one of the first applications of a next generation sensor network in international sport events, and it demonstrated the usefulness of the emerging sensor-based air monitoring technology in rapid network deployment to supplement existing air monitoring.

## 1. Introduction

Air pollution has been shown to have direct links to adverse health effects, even following short duration exposures [1,2,3]. Pollutants, such as nitrogen oxides, from combustion sources can increase the possibility of respiratory infections. Particulate matter (PM), either directly emitted or formed in the atmosphere, is also harmful, and PM_2.5_ (particle aerodynamic diameter < 2.5 µm) can penetrate deeply into the respiratory tract due to the small size. Long-term exposure to ozone (O_3_), a pollutant formed from oxides of nitrogen and hydrocarbons, can also reduce the lung function and produce symptoms of lung tissue inflammation [4]. Participants in sports, especially endurance athletic events, such as marathons, may be at special risk of adverse health outcomes, whether they are elite or amateur participants. They experience several health stressors in combination during events, potentially including heat, cold, rain and hills, adding to the stress of energy output to complete these events [5]. Air pollution may be an added stressor that may at times be present at levels endangering or at least irritating to the runners and possibly the spectators. Especially for runners, during exercise, air intake increases, and most air is taken through the mouth instead of the nasal system, which can filter some particles and gases [6].

Hong Kong hosts many athletic events, including a signature event, the Standard Chartered Hong Kong Marathon, called the Marathon hereafter. The Marathon route covers a range of communities and streets including the West Harbor Crossing (a long sub-bay tunnel) in the city. These places are often quite polluted by traffic from the on-road trucks, diesel buses and light duty vehicle. Various measures have been implemented during the Marathon to improve the route-specific air quality for participants, including traffic control, closure of the tunnel to traffic and switching on the mechanic ventilation for the tunnel. The temporary traffic control measures are expected to improve the air quality along the Marathon route. Event organizers and government co-sponsors were interested in gathering and communicating facts regarding air quality during the event. However, the existing network of regulatory air monitors was determined to be inadequate to indicate actual air pollution concentrations along the Marathon route due to the highly heterogeneous nature of air pollutants in the urban environment, and the impact of the change in local traffic may not be directly captured in urban background air quality [7]. There was a need for site-specific air monitoring to provide *in situ* air quality [8]. However, the short-term duration of the sports event makes the *ad hoc* deployment of traditional equipment difficult or not possible because of the limitations of power supply and space. 

Recently, there has been an emerging trend of using the next generation air sensors to provide highly resolved air quality data as an alternative monitoring option [9,10]. Due to their low cost, small size and low power consumption, the new technologies are very appealing for use in situations where traditional monitors are impractical [9,11]. The sensor-based systems have been used by researchers in different programs for fixed, mobile and community air monitoring applications [11,12,13,14,15,16,17]. However, some applications of these new approaches to environmental sensing still pose some technical challenges. Chief among these is that the pollution data reported by sensors and sensor systems require critical and careful evaluation under different ambient conditions [11]. Among the important factors known to impact the data from various low cost sensors, such as the ones we employed (electrochemical cells for CO and NO_2_), as well as for ozone, are positive and negative interferences with other common ambient co-pollutants, temperature and humidity impacts on reported pollutant responses, long-term drift and cell life time under extremely high and low humidity conditions. Further, it is apparent that cells for a given pollutant may be somewhat variable in response to the above factors. Investigation of how to provide robust calibration and to improve sensor data accuracy considering these factors has been performed by others [17,18]. These include development of simple linear regression fitting of observed data, multiple regression estimations performed to further improve the fits taking factors, such as humidity, temperature and co-pollutants into account. Finally, recent studies have applied neural networking protocols, where multiple factors are included [19,20,21,22]. 

The study presents our work in the development of a next generation air sensor system and its application in an *ad hoc* air monitoring network, in which the air sensors were placed and operated at the street level to monitor the air along the Marathon route in urban Hong Kong and the near-real time calculation and communication of health-indexed findings of air quality to the public. The deployment of the sensor network also provided a unique opportunity to investigate the impact of temporary traffic control measures on the local street level air quality in conditions that are somewhat more challenging than what has been evaluated in prior studies. The study also demonstrated the usefulness and potential of the air sensor-based monitoring approach for high temporal-spatial resolution air pollution measurement to meet environmental monitoring challenges.

## 2. Methodology

### 2.1. Sensor Platform Development

Several important design criteria were identified to produce an effective air sensing system for air monitoring along the Marathon route. These included the need for a system that was compact and light weight to enable easy field transport, to fit on a tripod and to allow on-site installation without infrastructure support; a high level of integration of sensors to measure the criteria of air pollutants, as well as temperature and humidity in real time; wireless data communication capability for real-time air quality broadcasting and battery power operation of at least 24 h for the extended remote operation in the field.

**Figure 1:**
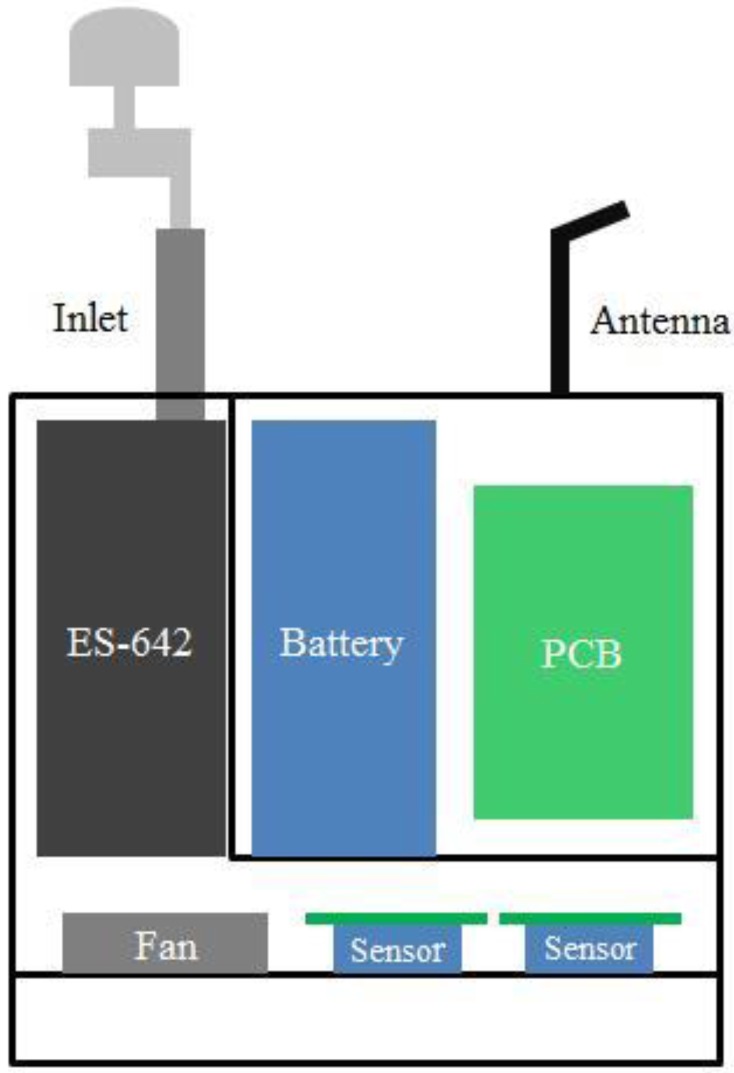
Schematic of the mini air station (MAS) system with different components.

The design and development of the monitoring system followed these criteria. Figure 1 shows the setup and internal view of the mini air station (MAS) system. The system is relatively small in size (25 cm × 16.5 cm × 29 cm) and light weight (10 kg). The main components and functions of the MAS are briefly described below and shown in Table 1:

- Two electrochemical sensors (NO_2_-B4 and CO-B4, Alphasense Ltd, Great Notley, UK), assembled on individual sensor boards supplied by the manufacturer, were selected for NO_2_ and CO gas measurements. The system was designed to host, at most, 6 sensors. To provide traffic-related criteria gas pollutants here, only two sensors were employed. The NO2 sensor was fitted by the manufacturer with an ozone filter to minimize the interference of the ozone on the sensor response. A laboratory test also confirmed little impact of ozone on NO_2_ sensor performance.
- A photometer (ES-642, Metone Ltd, Grants Pass, OR, USA) with a PM_2.5_ cyclone inlet was used for monitoring PM_2.5_ concentration. This photometer is equipped with a controlled, heated inlet to condition incoming air, but it was found to consume excessive battery power in the humid conditions found in Hong Kong. The heater was disconnected, and humidity correction was applied, as discussed later.
- A digital temperature and humidity sensor (SHT-25, Sensirion, Staefa, Switzerland), with a vendor-specified accuracy of ±1.8% for relative humidity (RH) and ±0.2 °C for temperature, was used to monitor the ambient environment and also to provide data needed to compensate their influence on the performance of the pollutant sensors.
- An Arduino micro MCU board (MEGA ADK, Arduino), mounted with a custom-made shield board with GPS and GSM modules, served to control basic communication functions for the systems: (1) data acquisition from the electrochemical sensors, the photometer, the humidity and temperature sensor and GPS; (2) data transmission from the location to a server at the lab of City University of Hong Kong; (3) data storage on an SD card was included to ensure data storage; (4) data display on a screen mounted on the case of the system.
- A 24-V 20-Ah lithium ion battery pack was used as the power supply for the system. This pack was capable of powering continuous operation of the entire system for a minimum of 24 h.

In addition to the NO_2_, CO and PM_2.5_ pollutants monitored by MAS, ozone was measured using portable ozone monitors (POM, 2B Technologies, Boulder, CO, USA), which are compact UV-based ozone monitors that have been used for portable ozone measurement [23]. Electrochemical cells are available for ozone measurements, but those available at the time of this test were found to be subject to considerable interference and artifacts of oxides of nitrogen in the humid atmospheres experienced in Hong Kong. Table 1 gives the technical specifications of the MAS system and POM.

**Table 1:** Specifications of NO_2_ and CO sensors, ES-642 and portable ozone monitor (POM).

Pollutant	Sensor	Sensitivity	Response Time	Measurement Range	Zero Drift
NO_2_	NO_2_-B4	−250 to −600 nA/ppm at 2 ppm NO_2_	<25 s from zero to 10 ppm NO_2_	0–20 ppm	0–20 ppb change/year in lab air
CO	CO-B4	420 to 650 nA/ppm at 2 ppm CO	<15 s from zero to 10 ppm CO	0–1000 ppm	<100 ppb change/year in lab air
PM_2.5_	ES642-PM_2.5_	0.001 mg/m^3^	NA	0–100 mg/m^3^	Automatic zero every hour
O_3_	POM	2 ppb	20 s for 100% of step change	2 ppb–10 ppm	<2 ppb/day

### 2.2. Sensor Performance

Extensive laboratory characterization and field evaluation of sensor and system performance were carried out prior to the MAS deployment in the Marathon event monitoring. The following section presents a brief description of the performance test procedures and a summary of both laboratory and field evaluation results.

#### 2.2.1. Laboratory Performance Tests

The laboratory performance test focused on three main components: (1) establishing the linearity and lower detection limit of the electrochemical sensors; (2) determining the impacts of humidity and temperature on sensors’ performance; and (3) calibration of POM units with standard ozone gas.

For the first test component, NO_2_ and CO sensors were set up in a Teflon chamber, and two calibrated gas analyzers were used as references, *i.e.*, the NO_2_ analyzer (Model T500U, Teledyne, Thousand Oaks, CA, USA) and CO analyzer (Model T300U, Teledyne). The gases with different concentrations (0, 20, 40, 60, 80, 100, 120, 160, 200, 240 ppb for NO_2_ and 0, 1, 2, 3, 4, 5, 6 ppm for CO) were used to test the linear response of the sensors. Prior to each test, zero air from a zero air generator (Model 701H, Teledyne) flushed the sensor chamber first for 15 min. The detection limits were determined by the following Equation (1). Ozone monitor (POM) calibrations were conducted using O_3_ gas generated from a calibration source (Model T700U, Teledyne) and flushed through the POM over a range of 0 to 300 ppb with steps of 50 ppb, Each step lasted 10 min. The selection of the concentration steps was to cover the range of typical urban pollutant concentrations. The detection limits (*DL*) for sensor evaluation are defined as:

(1) DL=3.3σ/S

where *σ* = the standard deviation of the sensor response at zero air and *S* = the slope of the calibration curve [24].

Ambient temperature and relative humidity have been reported to impact electrochemical sensors’ response to air pollutants, especially at a low concentration range in ambient monitoring applications [9,25]. Laboratory tests were carried out by supplying temperature- and humidity-controlled standard gas to the NO_2_ and CO sensors. Briefly, for humidity testing, the target gas concentration was generated from the dilutor and was blended with clean air from the zero air generator (Model T701H, Teledyne) that was pumped through distilled water with a controlled flow rate and supplied to the sensors with a relative humidity range of 40% to 70%, as determined by a SHT 25 temperature and humidity sensor. We were unable to generate higher humidity concentrations with this procedure. For temperature tests, the gases were passed through a Teflon tube coil submerged in a temperature-controlled bath system to deliver the test gas temperature in the range of 15–21 °C, typical ambient conditions in winter time in Hong Kong. The sensor responses to the varying temperature and humidity were recorded to derive the correction algorithm to compensate for their impact on concentration measurement.

#### 2.2.2. Field Performance Tests

**Figure 2:**
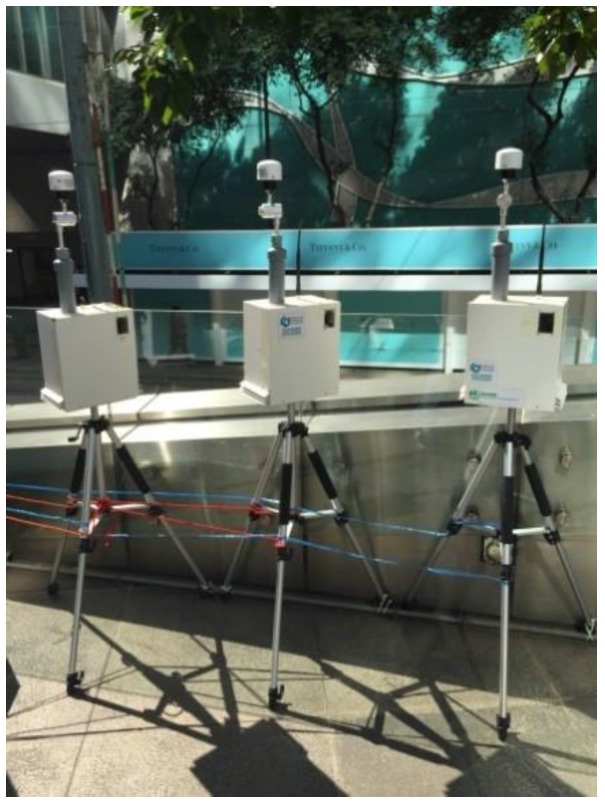
MAS field tests at the Central Air Quality Monitoring Station (AQMS), Hong Kong, China.

Prior to the deployment of systems in the Marathon, three identical sets of monitoring systems, mounted on tripods, were collocated with the roadside Air Quality Monitoring Station (AQMS) in Central, Hong Kong operated by the Hong Kong Environmental Protection Department [26], as shown in Figure 2. The station is located at the junction of Charter Road and Des Voeux Road with busy traffic. The PM_2.5_ inlets were 4.5 m above the ground. Field collocation tests were carried out from 16 January 2015 to 18 January 2015. During the test, the sensor systems were placed on the same platform at a distance of 1 to 2 m from the inlets of the AQMS. Closer proximity of the sensors to the AQMS inlets was possible and might improve the comparisons to some degree; however, ambient air monitoring siting practices call for case-specific separation of 1 or 2 m from regulatory-type PM_2.5_ monitors that run at 16.7 liters per minute. The setup used in this study was consistent with the accepted spacing practices, and the performance is further evaluated in the following section [27]. The sensor systems’ raw data were transmitted in real time to the cloud server located at City University at 5-s intervals. One-minute resolution AQMS data for PM_2.5_, NO_2_, CO and O_3_ were obtained from Hong Kong Environmental Protection Department (HKEPD).

#### 2.2.3. Correction Algorithms

Light scattering-based PM photometers for PM concentration measurements were subjected to two tiers of corrections, including the *k* factor to account for the photometer response to the concentration of particles with different characteristics from calibration aerosols [28] and the impact of relative humidity when above 40% due to the alternation of the particle refractive index by wetted particles [29]. The PM concentration can be obtained by:

(2) $$PM=PM_{reading}\times k\times (1+f\times RH^{2}/\left(1-RH\right)$$

where *PM_reading_* is the reading from ES-642, *k* is the mass correction factor, *f* is the humidity correction factor and *RH* is relative humidity. The field collocation test data from the PM sensor with the AQMS PM monitor were used as the necessary input to derive the k factor and the f factor in Equation (2).

For gas sensor correction algorithms, a mathematic formula in the form of Conc. = *f*(T,RH) × V + *f*(T,RH) was first derived from the laboratory test for NO_2_ and CO sensors to account for the impact of temperature (T) and/or relative humidity (RH), if any, on the sensor response (V) to the varying pollutant concentration (Conc.). The field collocation test data were then fitted in the formula with multiple regression to determine the individual coefficients. The further discussion of this fitting is presented in Section 4.2.

## 3. Sensor Network Development and Monitoring

### 3.1. Marathon Route Monitoring Sites

**Figure 3:**
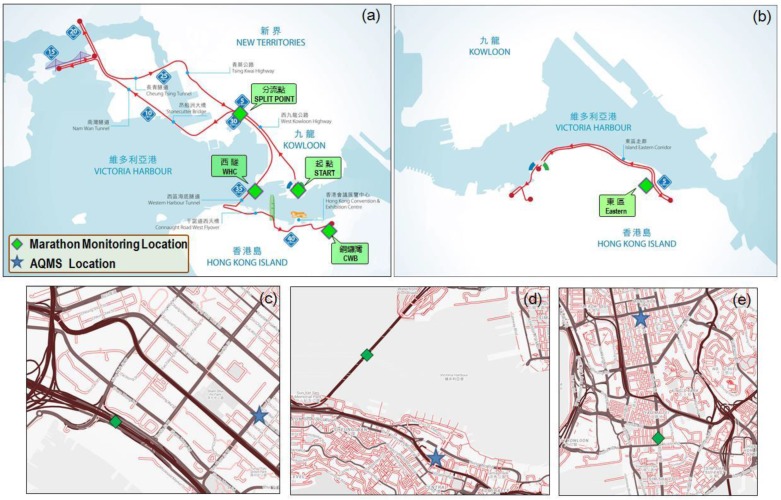
Air monitoring stations along: (**a**) the full/half marathon route; (**b**) the 10-km marathon route; the nearby AQMS run by HKEPD (**c**); Sham Shui Po AQMS near SSP; (**d**) Central AQMS near West Harbor Crossing (WHC); (**e**) Mong Kok AQMS near Tsim Sha Tsui (TST).

The Standard Chartered Hong Kong Marathon 2015 was held on 25 January 2015 from 05:30 to 13:00. The Marathon contains three routes, including the full marathon (42.2 km), half marathon (21.1 km) and mini-marathon route (10 km), as shown in Figure 3. The full marathon route follows main roads, highways, tunnels and bridges, covering commercial areas, residential areas and industrial areas of Hong Kong. Three sites along the full/half marathon route were selected for MAS deployment, as shown in Figure 3a:

- Start point in Tsim Sha Tsui (TST): The TST site (22°18'09.8"N 114°10'18.2"E) was close to the starting line of the marathon full/half route along Nathan Road, which is normally a busy urban street with a high traffic flow. The station was deployed on the curbside of the running route. The other side of roadway was traffic-controlled at different times during the event.
- Split point in Sham Shui Po (SSP): The SSP site (22°19'48.4"N 114°08'49.5"E) was the point where the full and half marathon route split. The station was located on the south curbside of a highway with adjacent lanes closed for the Marathon route, while there was constant traffic flow on the north side of the highway throughout the monitoring period. The distance between the MAS and north side traffic is about 12 m, and there was dominant offshore wind during the day; thus, the MAS site was upwind of local traffic emissions.
- West Harbor Crossing (WHC): The WHC site (22°17'41.8"N 114°09'03.5"E) was located at the middle point inside the tunnel of Western Harbor Crossing, which is a dual three-lane tunnel connecting Hong Kong island with Kowloon. The MAS was deployed on the curbside along the running route.

Additionally, two other sites on Hong Kong Island were selected for more complete coverage of the different marathon routes. For these two sites, the air quality data were directly obtained from the corresponding AQMS.

- Causeway Bay AQMS roadside point (CWB): The CWB site (22°16'48.0"N 114°11'07.3"E) was along the course;
- Eastern AQMS in Sai Wan Ho (Eastern Point, EP): The EP site (22°16'58.5"N 114°13'09.5"E) was at a 300 m distance from the 10-km route.

Figure 3–e also show the routine monitoring stations along the Marathon route. The routine monitoring stations of AQMS at Sham Shui Po (general station) and Mong Kok (roadside station) are 1.1 km and 2.2 km away from the corresponding MAS deployment sites of SSP and TST. On the Marathon day, sections of Nathan Road in TST where the MAS was deployed were blocked from 01:40 to 10:00, serving as the running route during the event. One side of West Kowloon Highway in SSP site was blocked from 00:45 to 11:30, while the other side was kept open for normal traffic. The tunnel crossing of WHC was blocked from 00:45 to 12:00. During the event, marathon service vehicles, such as police motorcycles, were permitted to run on the course. 

Our teams arrived at TST, SSP and WHC at around 02:00, 02:30 and 03:30, respectively. The MAS were powered on to assure stability of the electrochemical sensors for 24 h prior to the Marathon [25]. It took approximately 10 min to deploy the MAS at each site prior to the data transmission. The streaming of data to the designated server started from 03:00, 03:30 and 04:30 for the respective sites.

### 3.2. Green Marathon AQHI

The Air Quality Health Index (AQHI) is a health-risk-related indicator scaling from one to ten and 10+ developed in Hong Kong to provide information about health risks resulting from short-term exposure to air pollution as a basis to inform the public to adjust activity levels [30]. The AQHI is calculated as the sum of the percentage added health risk (%AR) of four key air pollutants: NO_2_, O_3_, SO_2_ and respirable suspended particulates (RSP or PM_10_) or fine suspended particulates (FSP or PM_2.5_), whichever holds a higher risk factor. The %AR of each pivotal pollutant depends on its concentration and a risk factor, which is derived from local health and air pollution data. Then, %AR is compared to a scale to obtain the corresponding banding of AQHI. The calculation follows the listed Equations (3)–(6) [31]:

(3) $$\%AR=\%AR\left(NO_{2}\right)+\%AR\left(O_{3}\right)+\%AR\left(PM_{2.5}\right)$$

(4) $$\%AR\left(NO_{2}\right)=[exp\left(\beta \left(NO_{2}\right)\times C\left(NO_{2}\right)-1\right]\times 100\%$$

(5) $$\%AR\left(O_{3}\right)=[exp\left(\beta \left(O_{3}\right)\times C\left(O_{3}\right)-1\right]\times 100\%$$

(6) $$\%AR\left(PM_{2.5}\right)=[exp\left(\beta \left(PM_{2.5}\right)\times C\left(PM_{2.5}\right)-1\right]\times 100$$

where $\%AR\left(NO_{2}\right)$, $\%AR\left(O_{3}\right)$ and $\%AR\left(PM_{2.5}\right)$ are the added health risk of NO_2_, O_3_ and PM_2.5_; $C\left(NO_{2}\right)$, $C\left(O_{3}\right)$ and $C\left(PM_{2.5}\right)$ are the hourly average of corresponding pollutants in units of micrograms per cubic meter (µg/m^3^); $\beta \left(NO_{2}\right)$, $\beta \left(O_{3}\right)$ and $\beta \left(PM_{2.5}\right)$ are added health risk factors; and for Hong Kong,

$$\beta \left({\text{NO}}_{2}\right)=0.0004462559$$

$$\beta \left(O_{3}\right)=0.0001393235$$

$$\beta \left(PM_{2.5}\right)=0.0002180567$$

The AQHI used for the Marathon was called AQHI-Green Marathon (AQHI-GM) and was calculated following the same rules. It was intended to provide near real-time air quality information for the participants during the race.

For the three MAS sites of TST, SSP and WHC, the systems provided the measurements of NO_2_, CO, PM_2.5_ and O_3_. SO_2_ was not included in the MAS monitoring, but hourly concentrations were obtained from the nearest regulatory monitoring stations for each site. For the PM component in the AQHI calculation, as we measured only PM_2.5_ in the sensor network, we assumed the same ratio of PM_2.5_/PM_10_ at each site with the nearest routine air monitoring station to derive the corresponding PM_10_ concentrations for %AR calculation. For the other two sites of CWB and EP, the AQHI was directly adopted from the corresponding AQMS stations.

## 4. Results and Discussions

### 4.1. Laboratory Performance Tests

**Figure 4:**
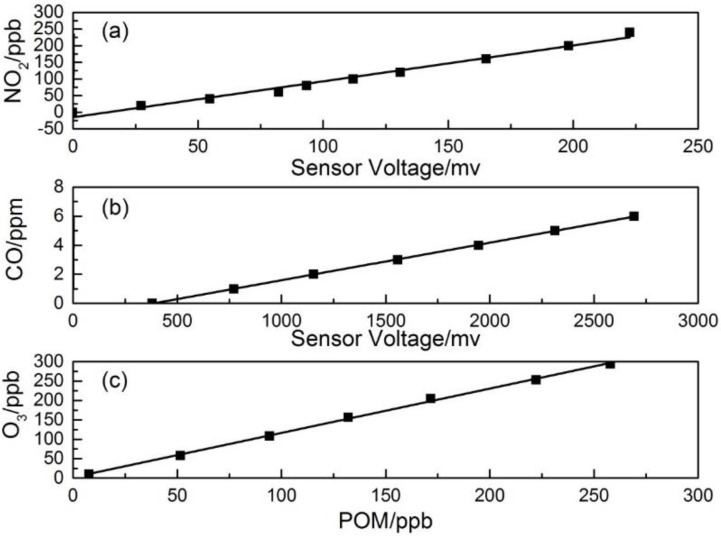
Linearity test results in dry gas conditions of (**a**) the NO_2_ sensor; (**b**) the CO sensor; (**c**) POM.

Figure 4 presents the multiple point calibration results for (a) a NO_2_ sensor, (b) a CO sensor and (c) a POM. As shown in the figure, both the NO_2_ and CO sensors and the POM demonstrated high linearity of sensor response to the pollutants in the concentration range, and R^2^ of the correlation is >0.99. The following Table 2 lists more laboratory calibration results of NO_2_ and CO sensors with the linearity equation and detection limit. The detection limits of both sensors are fairly low, as well as different factors, including the noise of the sensor signal processing circuit, the chemical stability of the electrochemical sensor electrolyte, *etc.* The low detection limits demonstrated in the laboratory study show their potential applicability for ambient air monitoring, but the performance evaluation is still needed within the range of pollutants’ concentration in applicable environments.

**Table 2:** Laboratory test results of NO_2_ and CO sensors and POM on dry gas.

Sensor	Equation	R^2^	Lower Detection Limit
NO_2_-B4 (NO_2_)	Y = 1.08X − 15.14	0.99	6 ppb
CO-B4 (CO)	Y = 0.0026X − 0.99	0.99	0.02 ppm
POM (O_3_)	Y = 1.14X + 2.06	0.99	4 ppb

Note: for NO2-B4, X is the sensor response voltage in mV, and Y is the concentration of the input NO_2_ gas in ppb; for CO-B4, X is the sensor response voltage in mV, and Y is the concentration of the input CO gas in ppm; for POM, X is the reading of POM in ppb, and Y is the concentration of the input O_3_ gas in ppb.

For temperature and RH tests, CO and NO_2_ sensors showed different behaviors and responses with varying conditions. In the temperature and RH range, CO sensor output showed no discernible variation at the same CO concentration. The correction formula was simplified as the equation found on dry gas, listed in Table 2.

For the NO_2_ sensor, the change of temperature showed little impact on sensor response. However, a positive relation was observed between the relative humidity and the gain of the sensor signal, possibly due to the humidity equilibrium between the ambient air and the sulfuric acid electrode. The mathematic correction algorithm for the NO_2_ sensor is formulated in the following Equation (7):

(7) $$Conc.=\frac{V-a\times RH-b}{c\times RH+d}$$

where *Conc.* is the corrected NO_2_ concentration in ppb; *V* is the sensor voltage response in mV; *a, b,* c and *d* are humidity correction factors.

The laboratory-test derived equations showed the inherent relation between the sensor gain in differential voltage and the pollutant concentration with the correction due to the varying temperature or relative humidity, if any. The parameters in the equation were further refined in the field performance tests by multiple regression analysis results between the actual pollutants’ concentration and the sensor gain under ambient conditions.

### 4.2. Field Performance Tests

**Figure 5:**
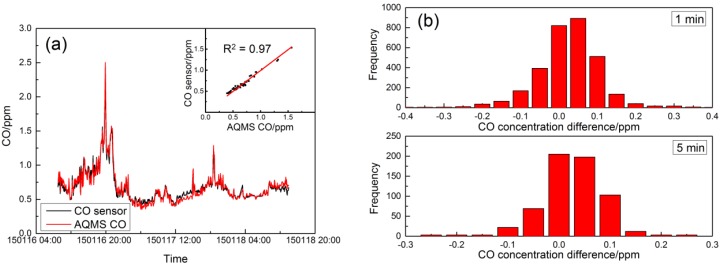
Comparison of the CO concentration from MAS and AQMS. (**a**) Time series of concentration; (**b**) histogram of the concentration difference in the 1-min and 5-min time average.

During the field test, a total of 3100 min of complete data were collected for the MAS. The ambient temperature and relative humidity were in the range of 15 to 22 °C and 33% to 89%, respectively, with temperatures similar to the range tested in the laboratory experiments, but with considerably higher humidity values. The one-minute averaged MAS and AQMS data were used to fit in Equations (2) and (3) to derive the coefficients for NO_2_ and PM_2.5_ concentration, but were also used to derive a linear relation for the CO sensor in ambient condition applicable to the range of ambient environmental conditions. The three sets of MAS systems in the collocation tests had consistent performance in their time series data of sensor gain output, and only one set of data is presented here. Table 3 lists the coefficients derived in the correction algorithms for one MAS system. Figure 5a shows the CO concentration from AQMS in comparison to the sensor data after correction. As shown in Figure 5a, the CO concentration was 0.68 ± 0.23 ppm (average ± standard deviation), and the two datasets had good agreement with a full reflection of the peaks and valleys by the sensor compared to the AQMS data. The inset shows the scatter plot of the one-hour CO concentration data between MAS and AQMS with a high linearity of correction and excellent agreement (Y = 0.91X + 0.06; R^2^ = 0.97). Figure 5b also shows the histogram of the difference between AQMS and MAS data. For the 5-min average, the mean error (ME) was less than 0.01 ppm, showing good agreement between the two datasets, while on the absolute term, the mean absolute error (MAE) was 0.05 ppm when the average of CO was 0.68 ppm during the period, and the mean relative error (MRE) was within 7% of the measured concentration.

**Table 3:** Correction equation for MAS sensors.

Pollutant	Equation	a	b	c	d
NO_2_	Conc. = (V − a × RH − b)/(c × RH + d)	10.97	−3.96	−0.17	0.33
CO	Y = aX + b	0.0025	0.099		
PM_2.5_	PM = PM_reading_ × *k* × [1 + f × RH^2/(1 − RH)]	0.75 (k)	0.25 (f)		

**Figure 6:**
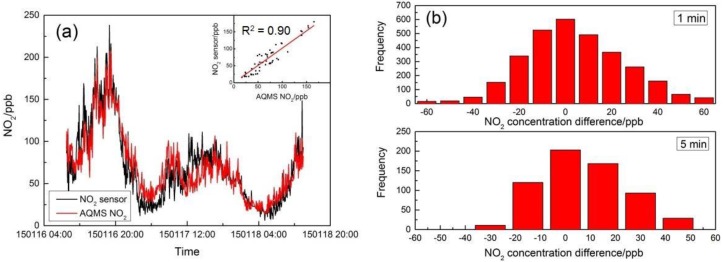
Comparison of the NO_2_ concentration from MAS and AQMS. (**a**) Time series of concentration; (**b**) histogram of the concentration difference in the 1-min and 5-min time average.

Figure 6a shows the field comparison results of AQMS NO_2_ data with MAS NO_2_ sensor data after application of the correction algorithm. Similar to the CO sensor performance, the time series data of the NO_2_ sensor and AQMS data followed very similar trends with good agreement. The oscillation of the NO_2_ concentration for both datasets appears to be attributed to the roadway diesel traffic plume pulses typically found in the roadside environment, instead of the sensor or instrument noise. The inset in Figure 6a shows that the correlation coefficient for the one-hour resolution data between MAS and AQMS is 0.90 with a slope of 1.09, and Figure 6b shows the statistics of the error on the 5-min average with a typical normal distribution, in which the mean error was less than 2 ppb, indicating the good overall agreement, but with both positive and negative errors. While the average NO_2_ concentration during the period was 69.8 ppb, the MAE was 14.1 ppb, and the MRE was 24% of the measured concentration.

**Figure 7:**
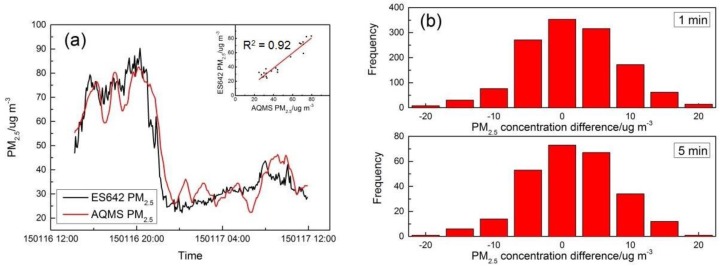
Comparison of the PM_2.5_ concentration from MAS and AQMS. (**a**) Time series of concentration; (**b**) histogram of the concentration difference in the 1-min and 5-min time average.

Figure 7a shows the MAS PM_2.5_ sensor performance compared to the AQMS PM_2.5_ data after k factor and humidity correction. At 5-min resolution, the two datasets showed good agreement in the overall trend with a correlation coefficient of 0.92 and a slope of 1.05. While the average PM_2.5_ concentration during the period was 46.9 μg/m^3^, histograms of the measurement error show the MAE was 5.5 μg/m^3^, and the MRE is 13% of the measured concentration. The field tests of MAS sensors in comparison with the routine AQMS monitoring data showed overall very good performance after correction by the developed algorithms in the temperature and humidity ranges encountered.

### 4.3. Air Quality along the Marathon Route

#### 4.3.1. Overview

On the Marathon day, ambient temperature and relative humidity were in the range of 16 to 22 °C and 40% to 80%, respectively, during the period of sensor network deployment along the Marathon route, similar to the ambient condition in the field test period. The AQHI among the 12 ambient monitoring stations and three roadside stations in Hong Kong was in the range of three to five and four to six, respectively, under the category of “Low” to “Moderate”, indicating the relatively clean regional background. The air quality in AQHI-GM along the Marathon route at five different locations, including the data collected from the MAS, is shown in Table 4. Although the MAS network sites of TST, SSP and WHC are located on the roadside or inside roadway tunnel environments, the AQHI-GM are generally equivalent or better than the roadside stations, suggesting the possible reduction of air pollution due to the traffic control before and during the Marathon event.

**Table 4:** AQHI-Green Marathon (GM) at the Marathon sensor network sites and at routine air monitoring stations. CWB, Causeway Bay.

Date and Time	Half/Full Marathon			10 km		General AQHI	Roadside AQHI
25 January 2015	TST	SSP	WHC	CWB	EP	Sham Shui Po	Mong Kok
03:00 to 04:00	4	NA	NA	5	4	4	5
04:00 to 05:00	4	4	NA	5	4	4	5
05:00 to 06:00	3	4	4	5	4	4	5
06:00 to 07:00	4	4	4	5	4	4	5
07:00 to 08:00	4	5	4	5	4	4	5
08:00 to 09:00	4	5	4	5	4	5	5
09:00 to 10:00	4	5	4	5	4	5	5
10:00 to 11:00	NA	5	4	5	4	5	5

NA: Data not available.

#### 4.3.2. Tsim Sha Tsui Site

**Figure 8:**
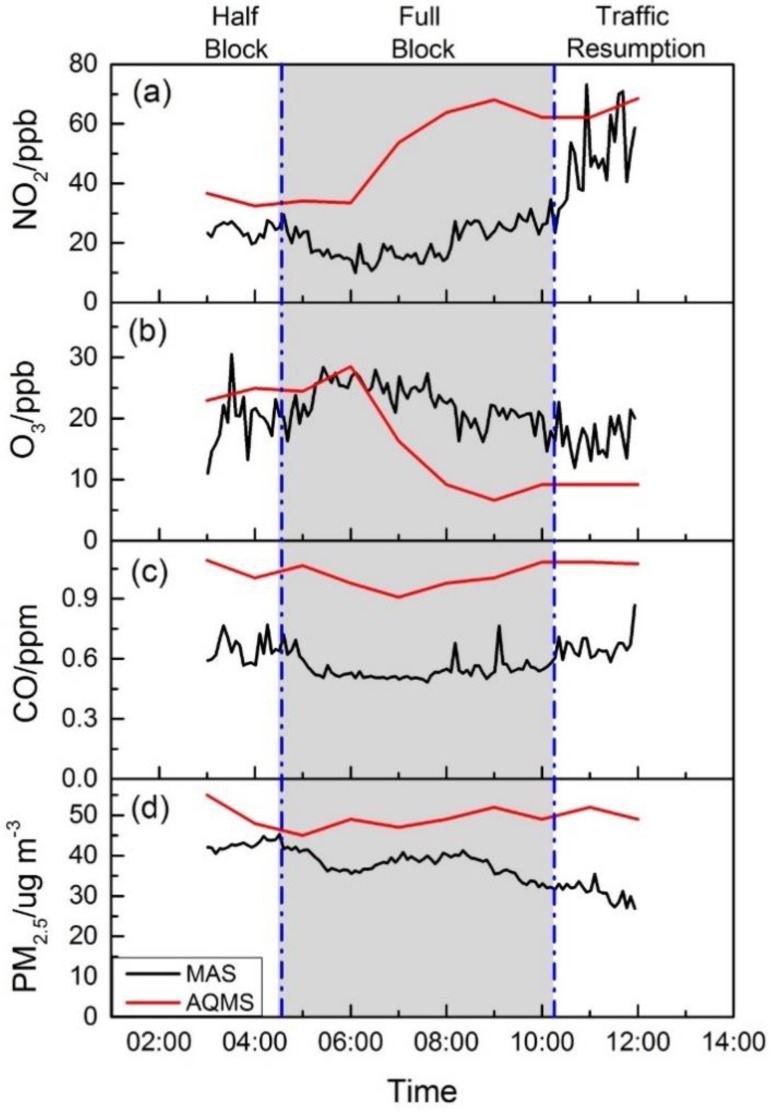
The pollutants’ concentrations before, during and after the Marathon at TST from MAS and at Mong Kok from AQMS. (**a**) Time series for NO_2_; (**b**) time series for O_3_; (**c**) time series for CO; (**d**) time series for PM_2.5_.

Figure 8 shows time series plots of NO_2_, O_3_, CO and PM_2.5_ concentrations at the start point at TST from MAS and the nearby roadside AQMS at Mongkok. The two sites are about 2 km in distance, separated by several street blocks. The AQMS in Mongkok showed a typical urban roadside site pollution profile with increased NO_2_ concentration during morning rush hour from 06:00 to 09:00. O_3_ concentration, however, decreased in the same time period, perhaps due to titration by local traffic-generated NOx pollutants. The TST site along the Marathon route, although also a roadside environment, had a contrasting pollution concentration profile due to the temporary traffic control measures implemented. As shown in Figure 8a, NO_2_ concentrations from MAS were close to the levels in roadside AQMS before rush hour started from 03:00 to 04:40 when one direction of the roadway was still open, and it remained flat all through the Marathon period until 10:00, with an average concentration of 20 ppb. Shortly after the traffic resumed at 10:00, the NO_2_ concentration increased quickly and reached an average concentration of 46 ppb, equivalent to the roadside AQMS concentration, clearly demonstrating the impact of traffic control measures on the street level air quality for NO_2_. As an additional possible consequence of traffic controls, as well as largely early morning hours of observation, O_3_ concentration also remained relatively flat throughout the Marathon period, with the lack of traffic inducing the NO pollutant in the surrounding microenvironment, as shown in Figure 8b. For CO, the major local contributors are petrol and LPG light bus and taxi traffic in Hong Kong for public transport [32,33]. Before the Marathon, when one roadway direction was still open with mostly private petrol vehicles and LPG bus and taxi, its concentration varied greatly with an average concentration of 0.65 ppm. During the Marathon, when the traffic was fully blocked from 04:40 to 10:00, the CO became more stable, with occasional peaks due to the passing-by patrolling motorcycles, as reported by on-site observers. The CO in the AQMS roadside site showed less variation with a slight increase during rush hours, while the levels are consistently higher than the Marathon site. PM_2.5_ showed a different pattern of concentration profile compared to the traffic-related gas pollutants of CO and NO_2_. The concentrations at both sites were at similar levels before the traffic at the Marathon site was fully blocked at 04:40, followed by a slight drop with lower concentrations that extended all through the Marathon period, while the traffic resumption showed no obvious impact on its concentration after 10:00. This appears to be consistent with other studies showing that the PM_2.5_ is characterized as a regional pollutant that is less affected by local traffic emissions [7].

#### 4.3.3. Sham Shui Po Site

**Figure 9:**
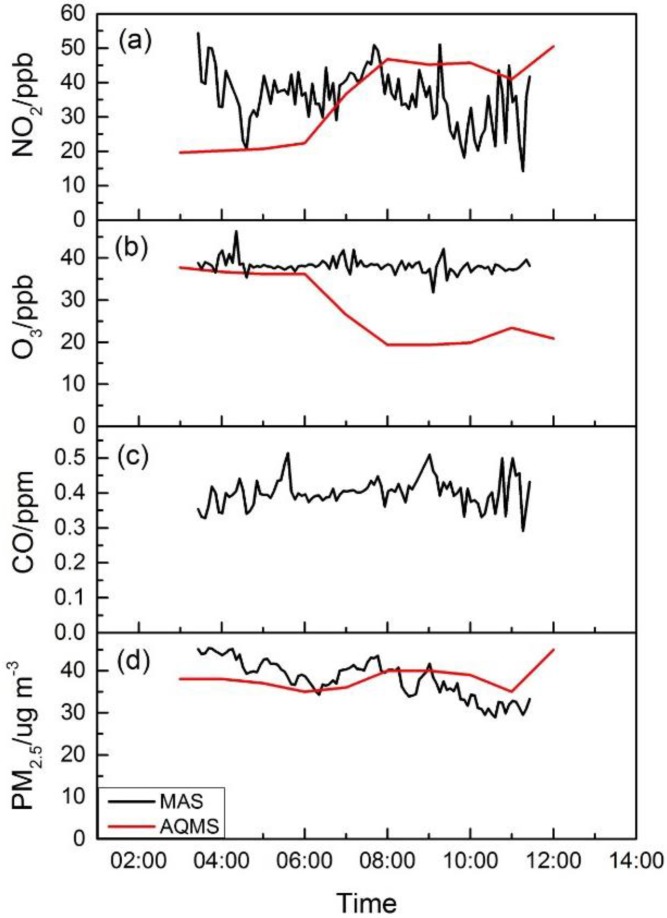
The pollutants’ concentrations before, during and after the Marathon at SSP from MAS and at Sham Shui Po AQMS. CO is not reported by the Sham Shui Po AQMS site. (**a**) Time series for NO_2_; (**b**) time series for O_3_; (**c**) time series for CO; (**d**) time series for PM_2.5_.

Figure 9 shows the NO_2_, O_3_, CO and PM_2.5_ pollutant concentration profiles for the SSP site with MAS along the Marathon route and the nearby AQMS in Sham Sui Po. The two sites were 1.1 km apart. The NO_2_ and O_3_ concentrations from the AQMS ambient site followed similar trends as the roadside site, as discussed in the previous section, showing the typical pattern of urban air quality. However, their concentrations at the Marathon site were relatively stable with little impact of the traffic, possibly due to the upwind positions relative to the roadway. CO concentration was in the range of 0.3 to 0.5 ppm at the Marathon site, with no significant variation, and the concentration is consistently lower than the TST Marathon site. The Marathon site was surrounded by urban traffic in the nearby street blocks, ranging from 0.5 to 0.8 ppm. No CO data were available from the AQMS at Sham Shui Po. For PM_2.5_, the two sites followed very similar trends with good agreement, further demonstrating that PM_2.5_ is more a regional pollutant with less spatial heterogeneity compared to other pollutants [34,35].

#### 4.3.4. West Harbor Crossing Tunnel

**Figure 10:**
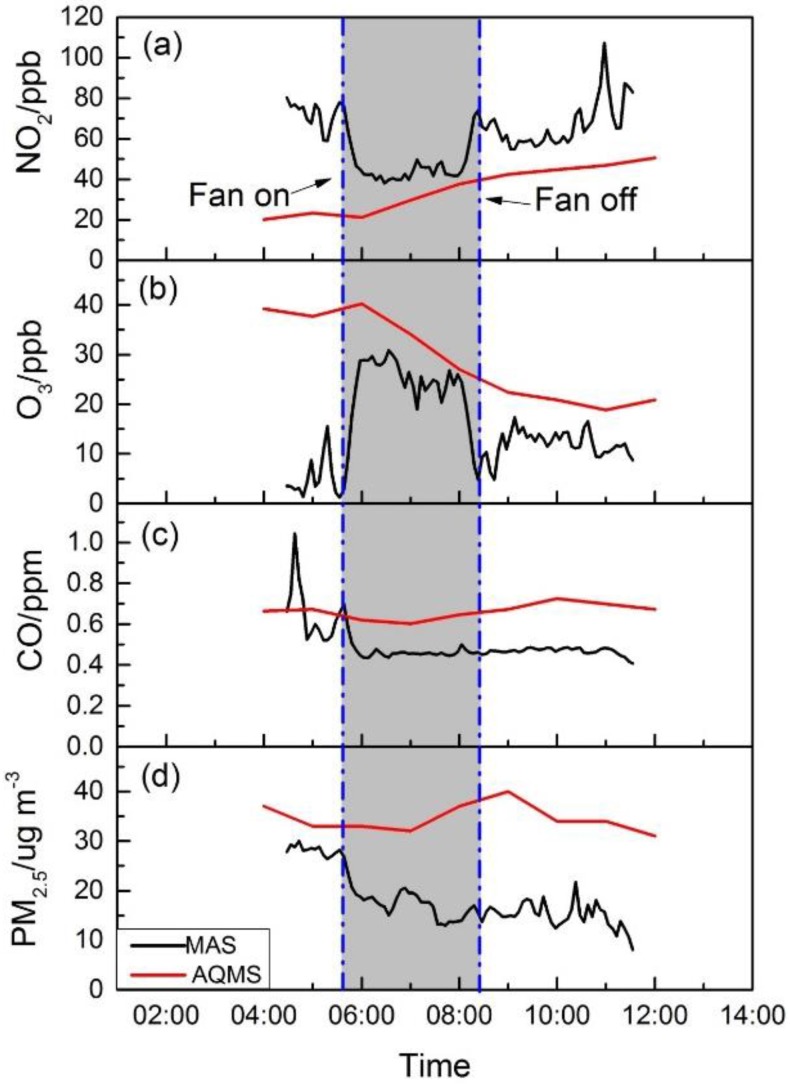
The pollutants’ concentrations inside WHC from MAS and at the Central AQMS. (**a**) Time series for NO_2_; (**b**) time series for O_3_; (**c**) time series for CO; (**d**) time series for PM_2.5_.

Figure 10 shows the pollutant concentration of NO_2_, O_3_, CO and PM_2.5_ in the WHC tunnel and from the roadside Central AQMS during the event. The Central AQMS, while quite a different site, was chosen for comparison due to its roadside traffic characteristics and central location in the roadway network on Hong Kong Island, which is connected to Kowloon by WHC. The NO_2_, CO and PM_2.5_ concentrations at Central AQMS showed typical urban pollution profiles in good agreement with those at the Mongkok and Sham Shui Po sites discussed in previous sections. Inside the unique microenvironment of the WHC tunnel, there were interesting observations in comparison to the other sites. NO_2_ concentrations were initially high at around 70 ppb, although traffic through the tunnel had been blocked since midnight. However, no active ventilation was performed. Once the active mechanic ventilation was initiated at 05:50, fresh outside air diluted tunnel air, and a clear decline of the concentration was observed; it remained low at around 40 ppb, until the fan was turned off, followed by an increase of the concentration. This may be explained by the additional formation of NO_2_ from the titration of remaining NO by ozone that was introduced into the tunnel during ventilation [36]. This can be seen from the ozone concentration profile in Figure 10b mirroring NO_2_, especially during the ventilation period, in which the ozone concentration increased and reached the ambient level. For CO and PM_2.5_, a clear decrease of concentrations was observed after ventilation started, and the concentrations remained low inside the tunnel, reflecting the absence of sources and limited passive ventilation conditions.

We also observed during the Marathon that the occasional diesel-powered service vehicles passing through the tunnel impacted local air quality with pulses of pollutants accumulating inside the tunnel. When NO_2_ levels between the three monitored sites are compared, it is clear that the tunnel site has two to three times the concentration. PM_2.5_ levels were also slightly higher in the tunnel, but were reduced by the ventilation operation, as well. It is likely that other vehicle-related harmful pollutants, such as black carbon, though not measured in this study, could be elevated, as well. This site was of concern to the race organizers based on participant complaints in prior year’s events. These observations suggest that runners in the tunnel could in fact be exposed to elevated pollutant levels if they entered during periods when pollutants from traffic stayed in the tunnel when ventilation was off. It might be useful to reduce or replace diesel-powered service vehicles with ones with a cleaner fuel, as well as to operate the mechanical ventilation system to reduce the runners’ exposure to air pollutants inside the tunnel.

## 5. Conclusion and Future Work

This study reports the development of a next generation air sensor-based monitoring system and its application for the international Marathon in Hong Kong. The successful *ad hoc* establishment of the route-specific air monitoring network and its real-time data broadcasting demonstrated the advantages of the next generation and compact air sensor system to meet the challenging needs in air monitoring that traditional air monitors are not likely to perform. One key demonstration from this study was to show how electrochemical and photometric sensors performed in the semitropical conditions of Hong Kong. We found that it is essential to characterize the concentration response to ambient environmental conditions. It is not possible to simply assume that these sensors can be plugged into a system and to expect that the data will be directly useful. With careful characterization of the sensor performance and quality control and assurance protocols, the sensing system was demonstrated to produce high quality, time-resolved data comparable in many respects to regulatory air monitors. There are factors that we did not address in this study that could prove important for deployment of electrochemical-based sensor systems. One very important factor is the possibility of response drift due to irreversible cell changes over time; this study represents its application over a short term with considerable data correction steps. Longer studies are needed to better understand sensors’ performance and limitations in the traffic impacted urban sites of Hong Kong and tropical/semitropical Asia. 

Through the route-specific air monitoring network, we found that temporary traffic control was effective in reducing the traffic-related air pollutant levels, for example NO_2_ and CO, while PM_2.5_ appeared to be less affected due to its regional nature. The mechanic ventilation of the tunnel was demonstrated to impact air quality inside the tunnel, and the use of cleaner fuel vehicles would have been suggested to help keep the air pollution levels low. Ozone in the tunnel, however, may be elevated due to the fresh air introduced through ventilation, and reactions in the tunnel may increase NO_2_ concentration. The results presented in this study provided an evidence-based case study to show the effectiveness of the temporary traffic control for the roadside microenvironment. However, the impact of traffic rerouting on other streets is uncertain. Further, longer term monitoring under differing siting conditions is necessary to demonstrate the nature of traffic impacted microenvironments in urban areas. The use of sensor-based monitoring platforms appear promising for such studies.
